# Potential Eligibility for Lecanemab in a Cohort of Alzheimer’s Dementia Patients from India

**DOI:** 10.1002/alz70859_106469

**Published:** 2025-12-26

**Authors:** Suvarna Alladi, Samim M Mondal, Faheem Arshad, Subasree Ramakrishnan, Sarath Govindaraj, Pallavi Nagaraj Sastry, Sandeep Kumar

**Affiliations:** ^1^ National Institute of Mental Health and Neurosciences [NIMHANS], Bengaluru, Karnataka India; ^2^ National Institute of Mental Health and Neurosciences, Bangalore, Karnataka India; ^3^ National Institute of Mental Health and Neurosciences, Bengaluru, Karnataka India

## Abstract

**Background:**

Lecanemab, a monoclonal antibody targeting amyloid, has been approved as disease‐modifying treatment for Alzheimer’s disease (AD) and is gradually gaining regulatory approvals across the globe. However, its accessibility and applicability in low‐and middle‐income countries (LMICs) remains limited due to constraints in biomarker availability, cost effectiveness concerns and underrepresentation of diverse cohorts in clinical trials. This study aimed to evaluate the eligibility of patients with Mild cognitive impairment (MCI) and AD from Cognitive Disorders Clinic (CDC) for treatment with lecanemab, based on clinical, and imaging criteria used in clinical trials.

**Method:**

A retrospective review of patients diagnosed dementia was conducted using data from the CDC registry. Patients with MCI and AD dementia were included based on the Lecanemab drug trial’s inclusion criteria. Cognitive assessments including Mini Mental Status Examination (MMSE), Clinical Dementia Rating (CDR) scales and MRI were reviewed. Eligibility was assessed by applying the appropriate use criteria for Lecanemab to all patients with MCI/AD. Plasma and Brain amyloid biomarkers were not available for the patients.

**Result:**

Of the 1448 patients with dementia seen in CDC from May 2016 to May 2021, 643 (44.4%) were clinically diagnosed with MCI (n=252, 17.4%)/AD (n=350, 24.2%). Applying the CDR criteria excluded 43 MCI and 165 AD cases. An additional 20 MCI and 10 AD cases were excluded based on age criteria. Applying the MMSE criteria excluded 28 MCI and 132 AD cases. Additionally, 18 MCI cases were excluded due to the presence of cerebral microbleeds. The primary exclusion factors included, advanced disease stage (40%), and contraindications such as significant comorbidities (30%) and microbleeds 102 (20%). Thus, out of the total 1,448 dementia patients, 134 (9.25 %) and of 643 patients with clinical AD or MCI, 134 (20.8%) were potentially eligible treatment with Lecanemab.

**Conclusion:**

A substantial proportion (80.75%) of dementia patients from LMICs may be ineligible for lecanemab due to diagnostic and resource limitations. However, 20.8% of clinically diagnosed AD could potentially be eligible with biomarker testing. Efforts to enhance biomarker availability, early detection, and cost‐effective strategies are crucial to enable equitable access to emerging AD therapies in LMICs.

